# Supplementary material for: Characterizing cell-type spatial relationships across length scales in spatially resolved omics data
Source: Nat Commun. 2025 Jan 3;16:350. doi: 10.1038/s41467-024-55700-1 (PMC11699133; doi:10.1038/s41467-024-55700-1)
Supplement: Supplementary file 3 — Supplementary Software 1 [file 41467_2024_55700_MOESM3_ESM.zip › CRAWDAD/plots/spleen/S9_spleen_cd4_near_folB_trends_heatmap.pdf]

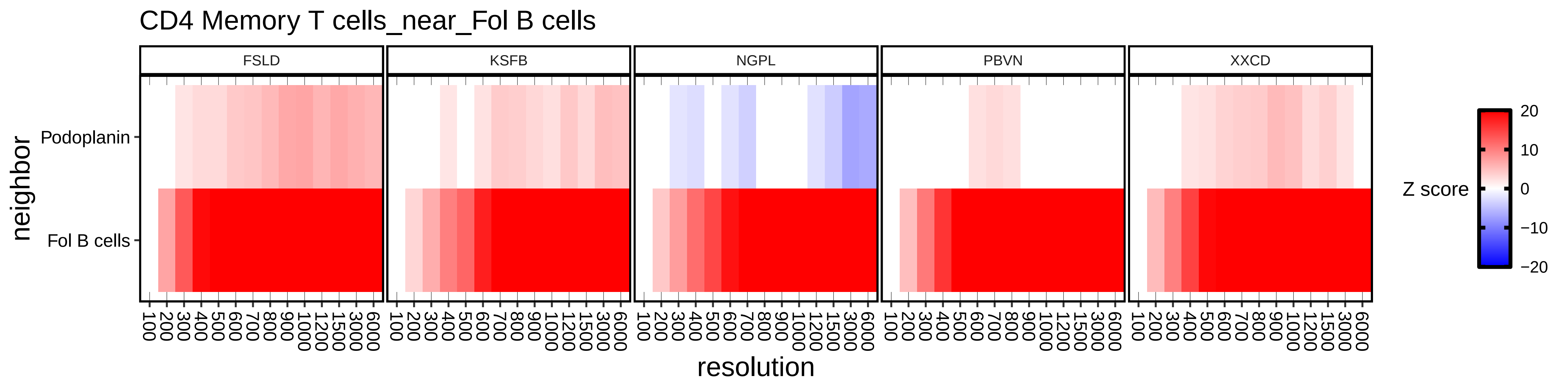
CD4 Memory T cells_near_Fol B cells
FSLD
KSFB
NGPL
PBVN
XXCD
neighbor
Podoplanin
Fol B cells
resolution
100
200
300
400
500
600
700
800
900
1000
1200
1500
3000
6000
Z score
20
10
0
−10
−20
